# Strategies towards Fully Recyclable Commercial Epoxy Resins: Diels–Alder Structures in Sustainable Composites

**DOI:** 10.3390/polym16081024

**Published:** 2024-04-09

**Authors:** Julio Vidal, Carlos Hornero, Silvia De la Flor, Anna Vilanova, Jose Antonio Dieste, Pere Castell

**Affiliations:** 1 Fundación Aitiip, Pol. Ind. Empresarium C/Romero, 12, 50720 Zaragoza, Spain; 2 Moses Productos S.L., Pol. Ind. Empresarium C/Romero, 12, 50720 Zaragoza, Spain; carlos.hornero@mosesproductos.com; 3 Department of Mechanical Engineering, Universitat Rovira i Virgili, Campus Sescelades, Av. dels Països Catalans, 26, 43007 Tarragona, Spain; silvia.delaflor@urv.cat (S.D.l.F.);; 4 GCR Group, Carrer Boters, s/n, 43717 La Bisbal del Penedes, Spain

**Keywords:** thermoset, Diels–Alder, recyclability, epoxy, covalent adaptable networks

## Abstract

The Diels–Alder equilibrium is a widely known process in chemistry that can be used to provide a thermoset structure with recyclability and reprocessability mechanisms. In this study, a commercial epoxy resin is modified through the integration of functional groups into the network structure to provide superior performance. The present study has demonstrated that it is possible to adapt the curing process to efficiently incorporate these moieties in the final structure of commercial epoxy-based resins. It also evaluates the impact that they have on the final properties of the cured composites. In addition, different approaches have been studied for the incorporation of the functional group, adjusting and adapting the stoichiometry of the system components due to the differences in reactivity caused by the presence of the incorporated reactive groups, with the objective of maintaining comparable ratios of epoxy/amine groups in the formulation. Finally, it has been demonstrated that although the Diels–Alder equilibrium responds under external conditions, such as temperature, different sets of parameters and behaviors are to be expected as the structures are integrated into the thermoset, generating new equilibrium temperatures. In this way, the present research has explored sustainable strategies to enable the recyclability of commercial thermoset systems through crosslinking control and its modification.

## 1. Introduction

Over the last century, polymers have emerged as one of the most frequently utilized, affordable, and streamlined materials. This is due to the wide range of properties that polymers have due to their diverse nature [1] and the dissimilarities in their chemical structures [2,3,4]. The disparities between the different types of polymeric materials begin with their origin, production, and processability, and they extend to their properties and end of life. Thermoplastic materials are more popular because of their superior performance in sectors such as automotive [5], where the reduction of the overall weight of vehicles is achieved by the incorporation of thermoplastic materials that substitute costly and difficult-to-recycle metallic elements, reaching stress values that can go up to 76 MPa [6]. On the other hand, thermoset materials are applied in more demanding sectors such as aeronautics [7], civil engineering [8], naval engineering [9], and other strategic sectors. Currently, their production represents approximately just 12% of the global plastic production [10]. However, they are projected to increase approximately 5% from 2023 through to 2032 [11]. Among thermosets, epoxy resins have shown to be one of the most promising structural materials [12], with growth exceeding 6% from 2021 to 2028 thanks to their efficient contribution to the green transition as light materials with high mechanical properties that are helping industries to reduce GHG emissions. In addition, comprehending the chemistry that underlies the resin’s structure has allowed us to adapt the material’s structure to meet the requirements of different processes while enhancing their characteristics [13] and application in potential sectors. Nevertheless, these thermoset polymeric structures cannot be efficiently recycled as their tridimensional stability does not allow for their reprocessability, and most of the materials are recycled by pyrolysis processes or landfilled.

The increasing industrial demands for high-performance materials, combined with the necessity of reducing the negative environmental impact of these materials, have generated interest in both research and industrial applications. Currently, there are two main areas of research: on the one hand, materials that withstand extreme conditions [14]. On the other hand, more sustainable materials can be developed by reducing the environmental impact by providing recyclability strategies to the material [15] or the use of more sustainable feedstock. These sustainability approaches, although compatible, are being handled separately. This specific research focuses specifically on environmental impact reduction through the adaptation of possible recycling methods, minimizing the negative environmental impact, and efficiently treating the materials after their end of life [16]. There are three main approaches [17] that break down the chemical structure of the thermoset [18]: (a) thermal, the pyrolysis [19] which is the only technology available on a commercial scale [20]. Its research is currently focussed on the process optimization for higher quality carbon fibers, the viability of the obtention of glass fibers, and the increase in quantity and quality of pyrolytic oils; (b) biological; enzymes are used to break down the thermoset structure into monomers or oligomers [21]; (c) chemical; use of solvents and chemicals as a strategy to reduce the polymer length, solvolysis [22,23], or a second chemical approach, modification of the thermoset structure to incorporate dynamic bonds [24]. The incorporation of dynamic bonds allows the thermoset to behave like a thermoplastic after the application of an external stimulus such as temperature. The trend towards new recycling methods is driven by the thousands of tons of thermoset materials that are about to reach their end of life and the need to avoid the current landfilling situation.

The inability to currently recycle commercially available thermoset materials [25] in an easy, scalable, and sustainable way is forcing the industry to improve recycling technologies. In fact, this impossibility is affecting the chemical industry by demanding eco-design thermoset materials [26], structures directly designed to be Recyclable, Reprocessable, and Repairable (3R) [27].

The 3Rs concept is delivered to the material through the incorporation of the so-called dynamic chemistry [28]. This allows them to be separated and regenerated on demand by the application of an external stimulus [29], such as temperature. There has been a great deal of research on the moieties that can generate dynamic mechanisms, either associative [30] or dissociative [31]. Some examples of the most common dynamic bonds studied to date are disulfide bonds [32], transcarboxylation [33,34], Diels–Alder [35,36], and imine bonds [37]. Thanks to their incorporation into the thermoset, the materials have a covalent bond that can be broken, allowing the polymer chains to flow under certain conditions and maintain their thermoset 3D complex polymeric structure under working conditions.

Among the different evaluated strategies, the Diels–Alder reaction has been widely applied for self-healing coatings and recycling purposes. The equilibrium between the diene/dienophile and the cyclohexene derivative is now being introduced into thermoset structures as a dynamic process. These moieties have some advantages over other dynamic structures. Firstly, as they are fully organic structures, it is often possible to obtain them from natural resources [33,38]. Although the biobased origin is not in itself an advantage, the current trend of the market towards fully biobased materials makes them interesting. Secondly, the variety of structures that can be anchored to each of the elements of the molecule allows the temperature of the reaction to be controlled.

As new structures and chemical groups are incorporated into the complex network of the thermoset, it is necessary to consider the secondary reactions even more, as the dynamic bonds break during the process, releasing various terminal groups. In the synthesis of the Diels–Alder adduct, the temperature must be properly controlled to avoid any parallel reaction that can take place due to the presence of other functional groups. For instance, at high temperatures, the Michael addition reaction [39] occurs through a nucleophilic addiction over an alpha–beta double bond. Therefore, the Michael reaction can act as a competitive reaction with the Diels–Alder in those cases in which the dienophile has a nucleophilic group attached, such as an amine. In this specific case, the dienophile with a nucleophilic group attached would act as a Michael donor and the diene as a Michael acceptor.

Incorporating dynamic systems, such as Diels–Alder, diamine, etc, in a composite and ensuring their correct reactivity is usually associated with the development and design of new thermoset systems. Therefore, the adaptation of resin reactivity to favor dynamic bonds is a long way that has only reached the market for self-healing purposes [40] but not for recycling.

The present study proposes a novel approach to provide novel and sustainable solutions for present thermosets and brings them one step closer to the market requirements and daily applications. Developments and research into their recyclability and their introduction into the circular economy [41] is a novel subject, and it also leaves plenty of room and opportunities for different technologies to help achieve the final goal: develop a recyclable resin. In this research, the Diels–Alder adduct has been introduced directly into a commercial thermoset system, upgrading its polymeric network with dynamic systems. In addition, compatibility and reactivity have also been studied. The introduction of the dynamic bonds will give the commercial thermoset structures a degree of recyclability that will allow the system to adapt and open the door to new recycling, reuse, and repair capabilities. These qualities have already been reached by thermoset structures made in the laboratory but are still out of reach for the materials currently in the markets. The capability to control the crosslinking level provided by the incorporation of dynamic bonds as a hardener enables the modification of the crosslinking level of the materials after their curing, facilitating end-of-life treatments.

## 2. Materials and Methods

### 2.1. Materials

The commercial epoxy resin used during the study for the formation of the final thermoset material is the Infugreen epoxy resin with the 4770-hardener, which is an amine-based crosslinker. Both components were purchased from MEL composites (Barcelona, Spain). The Infugreen 810 resin is a partially biobased resin commercially available, developed by Sicomin (Châteauneuf-les-Martigues, France) and marketed as GreenPoxy with a biobased percentage of biobased carbon of 29%, although in the epoxy resin alone it goes up to a 38% of plant-based carbon. The functional groups of both epoxy and hardener were measured following the ISOs 3001 [42] and 1877-2 [43], respectively. In this way, it was obtained that the SR 810 resin has 5.9 ± 0.1 mol of epoxy groups per kg of resin and the SD4770 has 10.3 ± 0.2 mol of amine groups per kg of commercial hardener and that for the commercial mixture, 100 g of Infugreen 810 reacts stoichiometrically with 29 g of hardener SD4770. Consumables used in the thermoset preparation were provided by MEL Composites (Barcelona, Spain).

The different chemical components N,N(1,3-phenylene) bismaleimide (CAS number: 3006-93-7) (BMI), furfuryl amine (CAS number: 617-89-0) (FAN), chloroform (CAS number: 67-66-3), and diethyl ether (CAS number: 60-29-7) have been purchased from Merck (Amsterdam, The Netherlands).

### 2.2. Chemical Synthesis of the Diels–Alder Adducts

For the synthesis of the Diels–Alder adduct used to transform the commercial thermoset material, the methodology described by Susana et al. [36] was used as a baseline. However, as the amine group in our proposed Diels–Alder moiety has higher reactivity than the alcohol used in the same identified research, it was necessary to perform several modifications in order to be able to synthesize the required moiety. These modifications of the described process were necessary to ensure the Diels–Alder reaction and to avoid the crosslink between BMI and FAN, which would result in impurities in the reaction or even become the predominant process. To avoid it, chloroform was introduced as a medium for the reaction.

**Figure 1:**
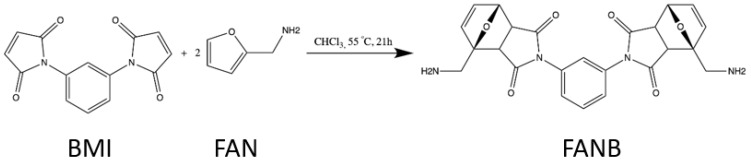
Scheme of the synthetic reaction carried out for the preparation of the FANB adduct incorporated into the thermoset structure.

Therefore, for the preparation of 8.4 g of the final product, from now on referred to as FANB (FAN + BMI) (Figure 1), 5.4 g of BMI was mixed with 3.5 mL FAN, using chloroform (200 mL) as a reaction solvent. The whole formulation was heated up to 55 °C, and it remained at this temperature for 21 h while being constantly stirred. Afterward, 120 mL of chloroform was evaporated; meanwhile, the rest was kept at 40 °C, and the product was precipitated dropwise into 300 mL of diethyl ether at 36 °C. During this last step, the stirring had to be very vigorous. Two hours later, it was filtered 2 times, and 30 mL of diethyl ether at 36 °C was added. Finally, the prepared product was kept in an oven at 40 °C for 24 h until it was fully dry. The product obtained showed the following 1H-NMR spectrum ^1^H-NMR (DMSO, 400 MHz, TMS, δ ppm): 8.49 (m, 4H, Ar), 6.46 (m, 4H, -HC=HC-), 4.93 (dt, 2H, HC-O-R), 4.36 (dm, 4H, -H_2_C-NH_2_), 2.92 (dm, 2H, HC-CO-), 2.38 (dm, 2H, HC-CO-), 1.32 (s, 4H, NH_2_). Endo and exo forms of the rings were present in the FANB molecule synthesized, confirmed by the signals at 4 ppm and those at 2 ppm.

### 2.3. Preparation of the Cured Materials

FANB adduct was introduced into the commercial formulation, which consists of 100 g of epoxy commercial resin and 29 g of hardener in order to prepare the samples. The FANB moiety was dissolved at room temperature in the epoxy resin, and afterward, the commercial hardener was added, and the whole mixture was stirred vigorously again. In this way, a maximum of 15% mol of FANB was introduced into the formulation, obtaining a homogeneous solution with no phase separation.

**Figure 2:**
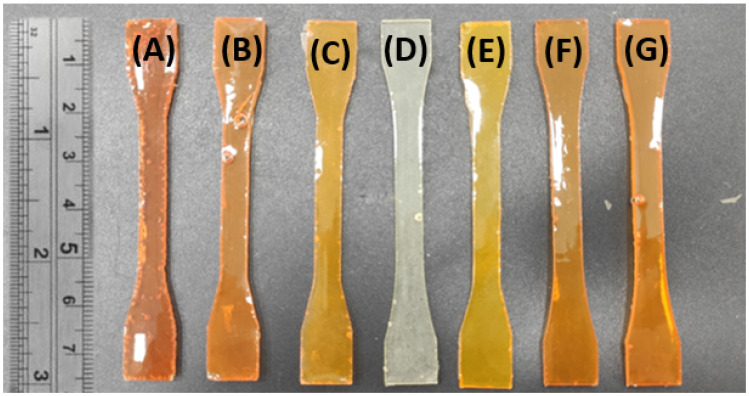
Picture of the specimens manufactured with different wt% of FANB: (**A**) amine excess 15 wt%; (**B**) amine excess 10 wt%; (**C**) amine excess 5 wt%; (**D**) commercial; (**E**) stoichiometric 5 wt%; (**F**) stoichiometric 10 wt%; and (**G**) stoichiometric 15 wt%.

The FANB can be added directly into the commercial system (Method 1), or the ratio between epoxy groups and amino groups can be kept by the reduction of commercial amino groups (Method 2). The specific masses used for the preparation of each sample with each of the methodologies applied can be found in Table 1, and images of the samples prepared can be seen in Figure 2.

Method 1: the FANB moiety was directly weighted and incorporated into the epoxy resin. In this way the number of amines in the final composition of the resin was increased, ensuring that all the epoxy groups have reacted with an amine, increasing the ratio of amino groups in the formulation. From now on, this methodology has been referred to as amine excess.

Method 2: the FANB was incorporated at different percentages, maintaining the same ratio of amine and epoxy groups as in the commercial resin system. This methodology is referred to as stoichiometric.

Once the dynamic structure was correctly mixed, specimens were prepared by a curing and post-curing process of 18 h at 80 °C and 5 h at 120 °C, which was fixed thanks to the curing results obtained by the DSC.

### 2.4. Characterization

The first step in the characterization of the different thermosets was the determination of their curing enthalpy. For this and for the measurement of the thermoset’s system Tgs, a METTLER TOLEDO DSC 3+ Star System (Columbus, OH, USA, EEUU) was used. In addition, for the study of the curing times, the isoconversional module of METTLER STARe System V17.00 was implemented as it allows to predict the reaction times at different temperatures thanks to its software’s nth-order kinetics models based on more than 3 dynamic conversion curves, by applying a constant Ea. The capability of the Diels–Alder adducts to cleave and regenerate the bond was also tested using the same differential scanning calorimetry (DSC) equipment and the methodology described in [36]. Samples in this analysis were prepared by dissolving the FANB moiety in acetone, which afterward was added to the commercial hardener. Acetone was evaporated before the epoxy resin was incorporated into the final mixture. Samples were stored at 4 °C until analysis. This methodology consists of a heating cycle where the material reaches 120 °C, and afterward, it is cooled down for 21 h at 60 °C, displacing the equilibrium towards the formation of the Diels–Alder cycle. The process was repeated several times.

To understand the kinetics of the reactions, the enthalpy of the curing processes was calculated by comparing the reaction at different heating rates (1 °C/min up to 20 °C/min). The Tg was measured by heating the cured samples from 5 to 160 °C at a rate of 20 °C/min. This measurement was performed three times in the same sample in order to ensure that the material was fully cured.

The thermomechanical properties were studied using a DMA Q850 (TA Instruments, New Castle, UK) equipped with a film tension clamp. Prismatic rectangular samples with dimensions around 30 × 5 × 2 mm^3^ were analyzed from −20 °C to 150 °C at 1 Hz, with 0.1% strain at a heating rate of 2 °C min^−1^. Two consecutive cycles were performed to ensure the completion of the curing process. For the relaxation analysis, tests were conducted using the same instrument and the film tension clamp on samples with the same dimensions as previously defined. After performing an oscillatory test in the same conditions as before, the samples were cooled down to 60 °C, and a constant strain of 1% was applied in static mode, measuring the consequent stress level as a function of time. The materials were tested only once at the selected temperature.

Finally, a spectroscope Bruker Alpha FTIR with Platinum-ATR was used to measure the cured samples with a resolution of 4 cm^−1^ by registering 24 scans from 4000 to 400 cm^−1^. For the thermal stability of the specimens, a Netzsch Libra II TGA was used (10 °C/min from 27 to 800 °C in an N_2_ atmosphere at 50 mL/min followed by an isotherm of 20 min at this temperature.

## 3. Results

### 3.1. Kinetic Studies

The first step for the incorporation of the synthesized Diels–Alder adduct into the thermoset system is to, on the one hand, evaluate its capacity to generate the adduct and, on the other, break it and form it again on demand. As a result, it was possible to observe that two different structures/diastereoisomers were generated.

The reactivity of the Diels–Alder groups has been widely used as a basis for the creation of dynamic networks in epoxy structures. In our study, three different thermal treatments were used to displace the equilibrium towards the formation of the adduct after its cleavage in the DSC. The Diels–Alder equilibrium was studied at 60 °C during three different time slots: 21 h, 12 h, and 0 h. These processes have allowed us to determine the time required to displace the reaction towards the cycle formation.

**Figure 3:**
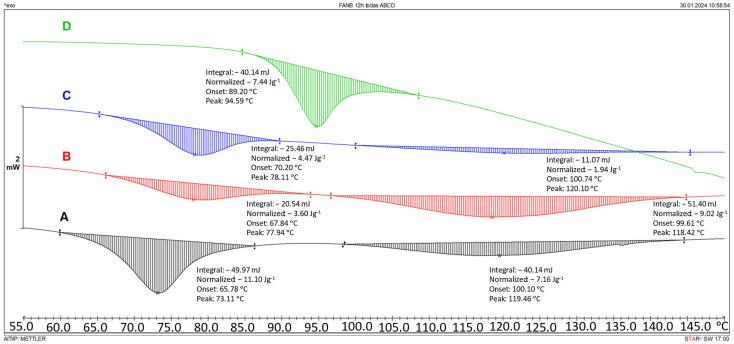
Diels–Alder reactivity cleavage with recovery cycles of 12 h at 60 °C; (**A**) after synthesis (black); (**B**) one cycle (red); (**C**) two cycles (blue); (**D**) three cycles (green).

Even though the system can be recovered during several hours at 60 °C, it is observable (Figure 3 and extended in the Appendix A) that the diastereoisomer generated during these equilibriums is mainly the endo (which is visible at lower temperatures, 73 °C) and therefore after several cycles of 21 h or two cycles of 12 h, the endo diastereoisomer is not measurable.

Once demonstrated, the capacity for the equilibrium was controlled through the application of an external stimulus, in this case, thermal treatments; the Diels–Alder adduct was incorporated into the thermoset system of the epoxy resin.

In this sense, two different methodologies were used (stochiometric and amine excess). The combinations achieved with the amine excess relation have presented a reduction of the curing time as the amount of adduct increases. However, increasing the number of amines by going over 10% of the adduct in weight does not reduce the reaction time, implying that the amines have reached the saturation point and become the limiting group. Hence, the reaction is limited by the amount and availability of the epoxy groups, as observable in the data shown in Table 2. Nevertheless, this effect is only visible at low temperatures, which could imply that another reaction, such as the Michael reaction, is taking place in the system at high temperatures.

On the other side, as the developed and proposed moiety substitutes the hardener used in the commercial systems in a stochiometric way, the reaction times for the complete curing process are hugely increased. This increment in the curing time of these samples demonstrates the lower reactivity of the new hardener incorporated into the system.

In this way, it was calculated that the curing temperatures could be similar. Nevertheless, the time for curing the formulations greatly varies depending on the amount of FANB, and it is even more dramatic as the amount of commercial hardener drops (Table 2).

Once the thermoset was prepared, the Tg of all these materials was measured (Table 3) to understand the effect that the incorporation and substitution of these adducts have on the system in each of the scenarios proposed: amine excess, where FANB is added over the commercial resin and therefore increasing the ratio of hardener in the final formulations, or stoichiometric, where different amounts of FANB are introduced and the respective commercial hardener is removed maintaining the same ratio of epoxy resin–hardener than in the commercial case.

It is possible to observe in Table 2 the lower reactivity of the amines provided by the FANB in comparison to those of the commercial system. This difference in the reactivity impacts the crosslinked structure and its thermal stability (Tg). As demonstrated by the Tgs measured by DSC (Table 3) and by DMA (Table 4), the differences between the stoichiometric and the amine excess methodologies are significant. In both cases, it is possible to observe that the presence of the adduct affected the reactivity and the final thermal properties of the materials prepared, i.e., the Tg. When FANB is added over the commercial formulation (amine excess), the Tg values are reduced less than in a complete substitution, demonstrating a bigger change in the thermoset structure for the first method (amine excess) than the second method (stoichiometric).

The differences in the Tg between the stoichiometric and amine excess samples are due to the incorporation of the FANB into the thermoset structure. In the case of the amine excess samples, the adduct does not interlink with the rest of the network, therefore affecting the chemical structure and the properties of the material less. In fact, it is possible to see that the FANB is not totally forming part of the thermoset structure, as shown in the comparison of the times calculated for the curing of stoichiometric and amine excess samples (Table 2) and in the Tg observed in the DSC (Table 3). For the stoichiometric case, bigger differences are observable as more adduct is incorporated, whereas in the amine excess material, the differences are not so relevant.

As in the DSC, it was not possible to differentiate the effect of the Diels–Alder reaction taking place during the heating process; DMA tests were performed to detect the separation between diene and dienophile through the storage modulus and tanδ evolution with temperature in an oscillatory test.

### 3.2. Mechanical Analysis

**Figure 4:**
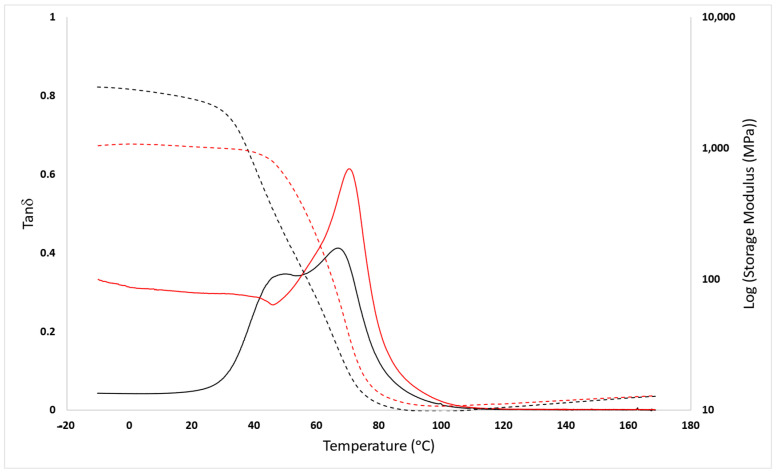
DMA analysis on the sample amine excess 10% wt FANB. Black continuous line: Tanδ of the first heating cycle; black discontinuous line: Storage Modulus of the first heating cycle; red continuous line: Tanδ of the second heating cycle; red discontinuous line: Storage Modulus of the second heating cycle.

In the DMA studies, the Diels–Alder separation is observable in the shape of the tand evolution with temperature, with a pre-transition (shoulder) at 50.4 °C that appears just before the a-relaxation (Tg value around 67 °C) (Figure 4, further representations can be found in the Appendix A). This process is only observable during the first heating. Therefore, it can be directly related to the separation of the Diels–Alder moiety and its inability to recover the cyclic structure without a thermal treatment. The cleavage of the D-A adduct in the thermoset structures implies the reduction of the crosslinking, and consequently, a change in the storage modulus values can be observed between the first and the second heating process.

Due to the temperatures at which the moiety is breaking, it is possible to conclude that the predominant diastereoisomer structure taken by the adduct within the thermoset structure is the endo.

As it was observed, the Diels–Alder moiety can be broken through the effect of the temperature, but it takes a specific process to regenerate it. For that reason, it was decided to carry out relaxation tests.

**Figure 5:**
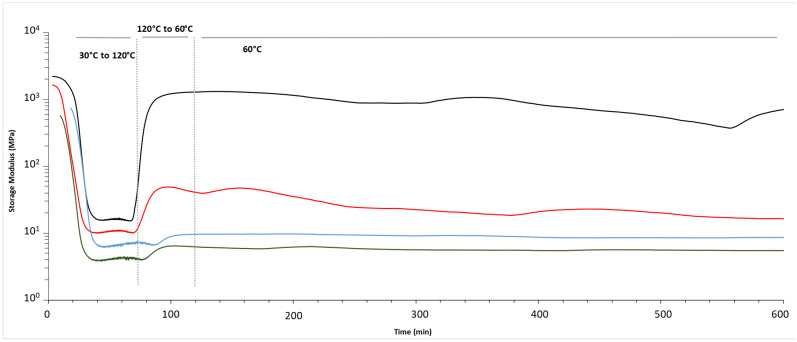
Stress relaxation analysis of the stoichiometric samples (black—commercial resin; red—5 wt% FANB; blue—10 wt% FANB; green—15 wt% FANB).

The relaxation tests were performed only on the stoichiometric samples because the amine excess incorporation of the Diels–Alder moiety was just physical and not chemical. In those cases, the Tg was not affected by the presence of the adduct, and the reaction times were quite similar to the commercial ones. Both effects lead to the conclusion that the stoichiometric samples were the ones with the adduct properly introduced in the system and, therefore, the ones that could show its reversibility (Figure 5).

On the relaxation tests, it is observed that the storage modulus of the materials decreases as the Diels–Alder bond is broken and the Tg is overpassed. However, as the temperature drops to 60 °C and it is maintained in time, the mechanical properties of the systems with the adduct are not fully recovered as they happen on the commercial material (Table 5 and Figure 5). Thus, the presence of the Diels–Alder facilitates the recyclability of the compounds by reducing the crosslinking density.

### 3.3. Structural Characterization

**Figure 6:**
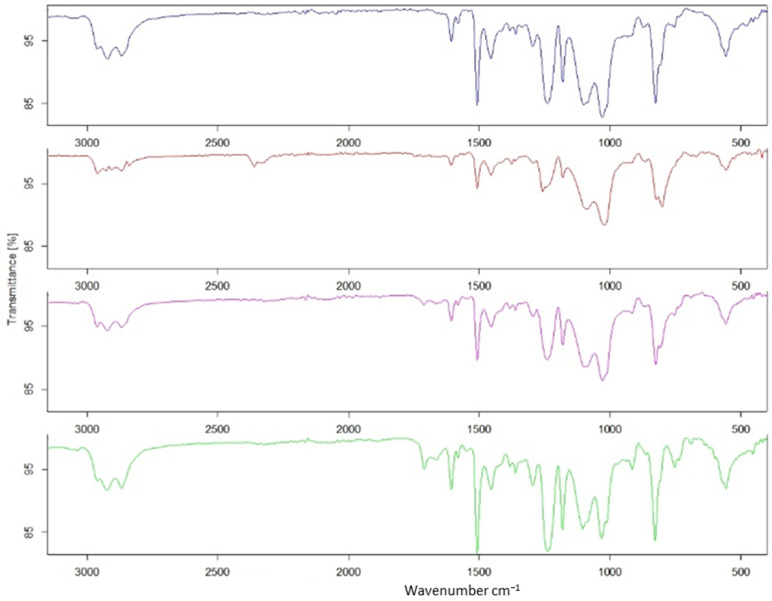
FTIR spectrum of the epoxy resin samples and of the additive formulations (black—commercial; red—5 wt% FANB; purple—10 wt% FANB; green—15 wt% FANB).

To better understand the effects of the adducts on the chemical structure, the stoichiometric samples were analyzed by FTIR. Figure 6 shows the differences among all samples prepared. In the spectra, the most relevant peaks related to the FANB and epoxy resin structure are visible. In all cases, the peaks at 910 or 860 cm^−1^, related directly to the epoxy group [44], were noticeable, and the same situation for the peak on 1250 cm^−1^ related to the asymmetrical aromatic C-O bond [45], showing complete curing of the functional groups. The analysis performed for all samples showed no relevant peak modification in terms of intensity and position.

**Figure 7:**
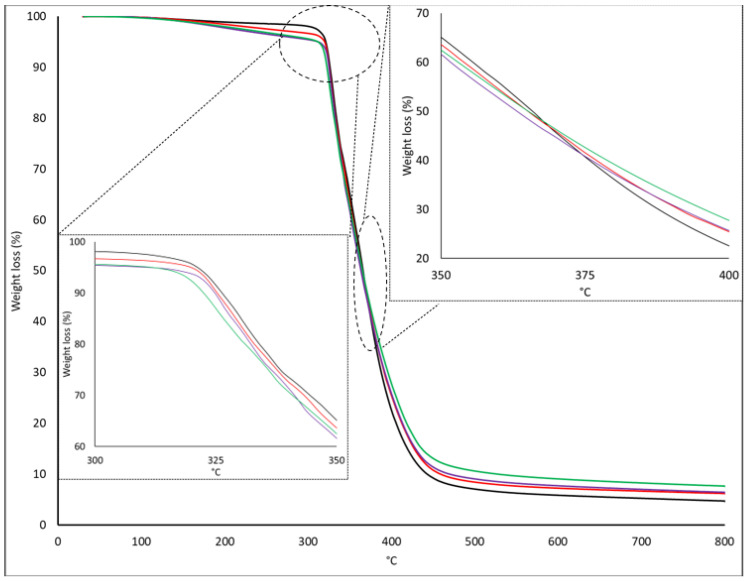
Thermogravimetrical analysis of the stoichiometric samples (black—commercial; red—5 wt% FANB; purple—10 wt% FANB; green—15 wt% FANB).

The differences observed on the stoichiometric samples during the relaxation DMA tests are shown in Table 5, together with the Tg values obtained in Table 3 and Table 4, and the curing kinetics shown in Table 2 reflect that there are changes in the structure of the thermoset samples, although the FTIR spectroscopy does not show any chemical change. It is for this reason that a TGA analysis of the specimens was performed. In this way, the effect of the moiety on the thermal stability was measured. In Figure 7, the presence of the adduct on the thermoset structure generates an increment in the ashes content of the sample that goes from around 6% up to the range of 10% for all samples with 10 and 15 wt% of FANB. The amount of adduct also has an impact on the onset temperature; as the amount of Diels–Alder functional groups increases, the onset temperature of the cured thermoset decreases from 311 °C on the commercial formulation down to 296 °C on the stoichiometric 15 wt% FANB, showing a lower thermal stability for the systems in which it is incorporated, which is shown in Figure 7.

## 4. Conclusions

The Diels–Alder reaction is a feasible approach to provide the thermoset structures with dynamic behavior that could facilitate their recyclability. As demonstrated in this study, the Diels–Alder moieties can be efficiently incorporated into the structure of commercial epoxy resins. The integration of these functionalities into the network generates new chemical bonds, which maintain the same nature as those used in the commercial formulation while providing a system that allows the modification of the crosslinking of the thermoset. The obtained results demonstrate the proper integration of the proposed moiety into the thermoset structure, maintaining the equivalent amount of epoxy groups and amino groups, generating crosslinked structures with similar mechanical performances.

Through this methodology, it has been demonstrated that it is feasible to integrate an external adduct on the thermoset network of a commercial resin system to modify its behavior. However, these modifications have an impact on the resulting thermal and mechanical properties of the final formulations that had been determined for all compositions developed. This strategy paves the way to develop sustainable and recyclable composite structures in a fast way through the modification of the commercial systems via Diels–Alder adducts. The controlled effect of the moieties will have a significant impact on their crosslinking degree and, therefore, on the recycling methodologies that can be implemented.

## Figures and Tables

**Table 1:** Weights used for each of the different formulations and the percentages of those components in the overall sample.

		Epoxy		Hardener		FANB vs. Epoxy	
		g	% (wt)	g	% (wt)	g	% (wt)
Stoichiometric	Commercial	15.50	77.50	4.50	22.50	-	-
	5% FANB	15.50	76.02	4.13	20.26	0.76	3.73
	10% FANB	15.51	74.57	3.75	18.03	1.54	7.40
	15% FANB	15.50	73.15	3.37	15.90	2.32	10.95
Amine excess	5% FANB	15.51	74.64	4.49	21.61	0.78	3.75
	10% FANB	15.50	71.96	4.49	20.84	1.55	7.20
	15% FANB	15.51	69.52	4.48	20.08	2.32	10.40

**Table 2:** Time in minutes that it takes for each formulation to reach 99% conversion at three different temperatures, determined by DSC.

		99% Conversion at 60 °C (min)	99% Conversion at 80 °C (min)	99% Conversion at 100 °C (min)
Stoichiometric	Commercial	389	123	43
	5% FANB	441	128	42
	10% FANB	5850	394	62
	15% FANB	14,600	1009	93
Amine excess	5% FANB	329	107	42
	10% FANB	262	82	35
	15% FANB	272	77	27

**Table 3:** Values of the Tg determined by DSC for all studied formulations.

Stoichiometric				Amine Excess		
Commercial	5% FANB	10% FANB	15% FANB	5% FANB	10% FANB	15% FANB
86.7	66.4	54.9	50.0	72.2	80.4	82.6

**Table 4:** Temperatures of the Diels–Alder cleavage and Tg determined by DMA.

Tanδ Peaks		1st Measurement		2nd Measurement	
		Diels–Alder (°C)	Tg (°C)	Diels–Alder (°C)	Tg (°C)
Stoichiometric	Commercial	-	71.8	-	74.1
	5% FANB	57.4	65.7	-	76.2
	10% FANB	47.4	61.9	-	63.8
	15% FANB	45.0	59.2	-	53.2
Amine excess	5% FANB	61.4	75.8	-	78.0
	10% FANB	50.4	67.0	-	70.5
	15% FANB	51.8	70.9	-	72.8

**Table 5:** Storage Modulus (MPa) of each formulation at selected temperatures and Tensile Modulus (MPa) during overnight relaxation at 60 °C.

		Storage Modulus at 30 °C (MPa)	Storage Modulus at 60 °C (MPa)	Storage Modulus at 120 °C (MPa)	Tensile Modulus at 60 °C (MPa) *
Stoichiometric	Commercial	2210	1341	16	1310
	5% FANB	1635	135	11	49
	10% FANB	732	13	7	10
	15% FANB	568	11	4	6

* Measured at isotherm 60 °C to measure the relaxation of the material.

**Figure A1:**
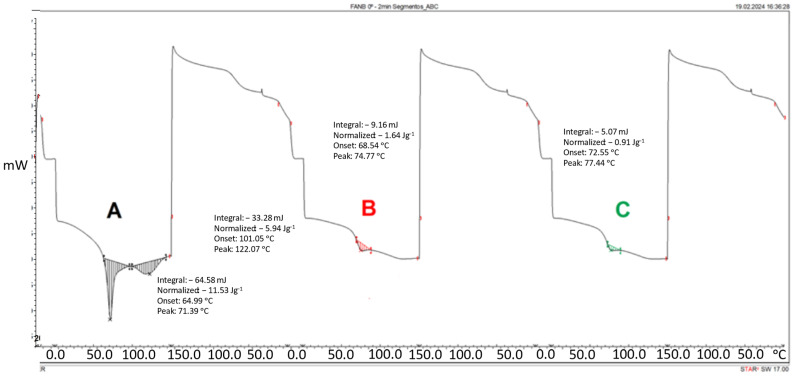
Diels–Alder reactivity cleavage without recovery processes; (**A**) after synthesis (black); (**B**) one cycle (red); (**C**) two cycles (green).

**Figure A2:**
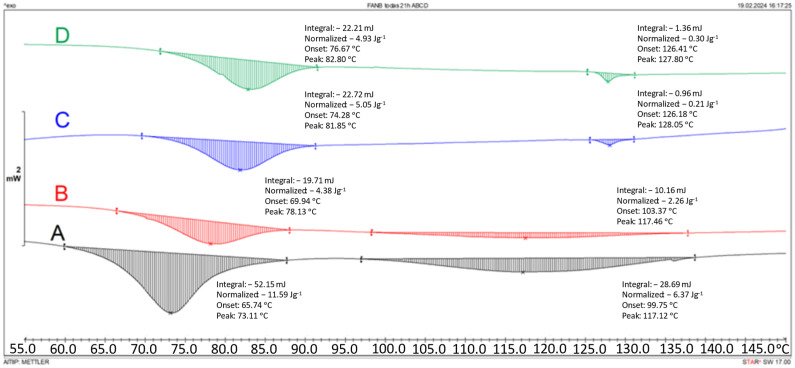
Diels–Alder reactivity by using relaxation processes of 21 h at 60 °C; (**A**) after synthesis (black); (**B**) one cycle (red); (**C**) two cycles (blue); (**D**) three cycles (green).

**Figure A3:**
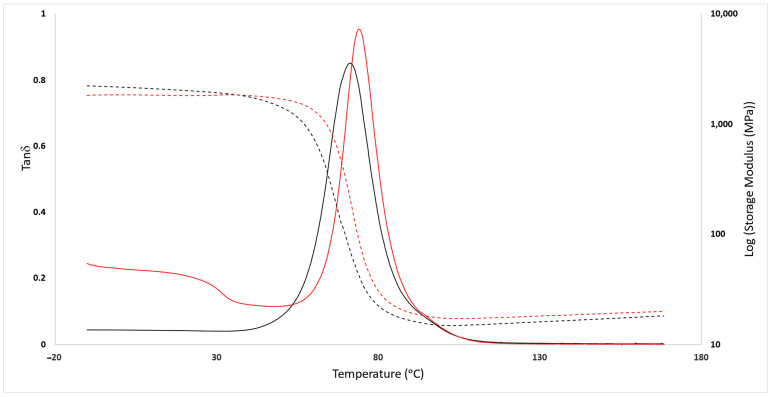
DMA analysis on the commercial formulation. Black continuous line: Tanδ of the first heating cycle; black discontinuous line: Storage Modulus of the first heating cycle; red continuous line: Tanδ of the second heating cycle; red discontinuous line: Storage Modulus of the second heating cycle.

**Figure A4:**
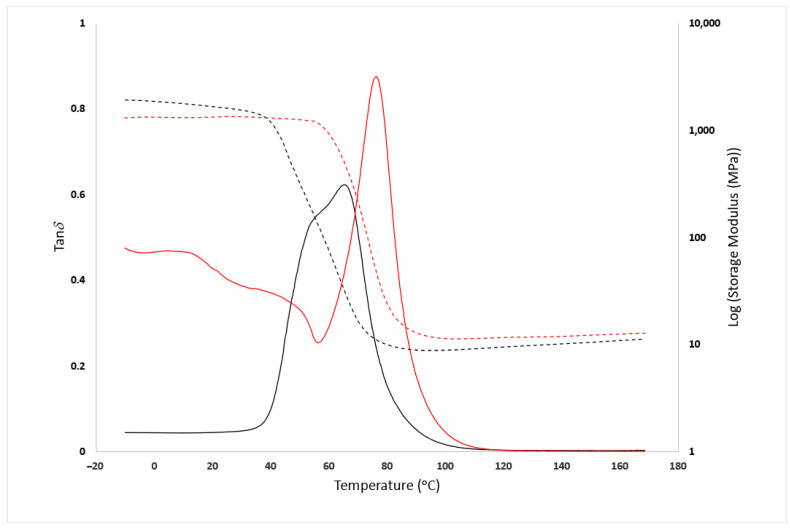
DMA analysis on the sample stoichiometric 5%wt FANB. Black continuous line: Tanδ of the first heating cycle; black discontinuous line: Storage Modulus of the first heating cycle; red continuous line: Tanδ of the second heating cycle; red discontinuous line: Storage Modulus of the second heating cycle.

**Figure A5:**
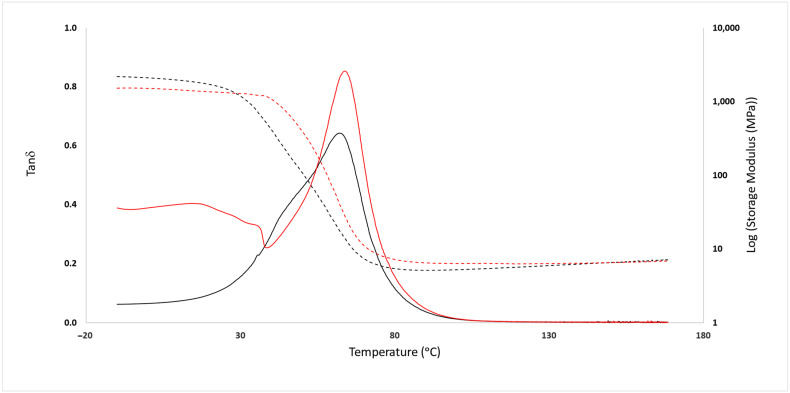
DMA analysis on the sample stoichiometric 10%wt FANB. Black continuous line: Tanδ of the first heating cycle; black discontinuous line: Storage Modulus of the first heating cycle; red continuous line: Tanδ of the second heating cycle; red discontinuous line: Storage Modulus of the second heating cycle.

**Figure A6:**
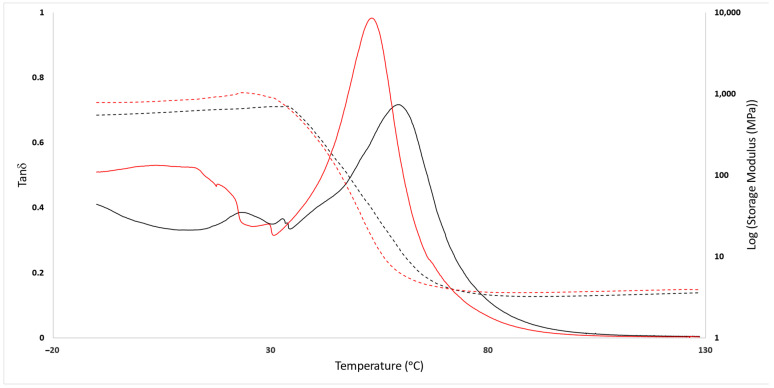
DMA analysis on the sample stoichiometric 15%wt FANB. Black continuous line: Tanδ of the first heating cycle; black discontinuous line: Storage Modulus of the first heating cycle; red continuous line: Tanδ of the second heating cycle; red discontinuous line: Storage Modulus of the second heating cycle.

**Figure A7:**
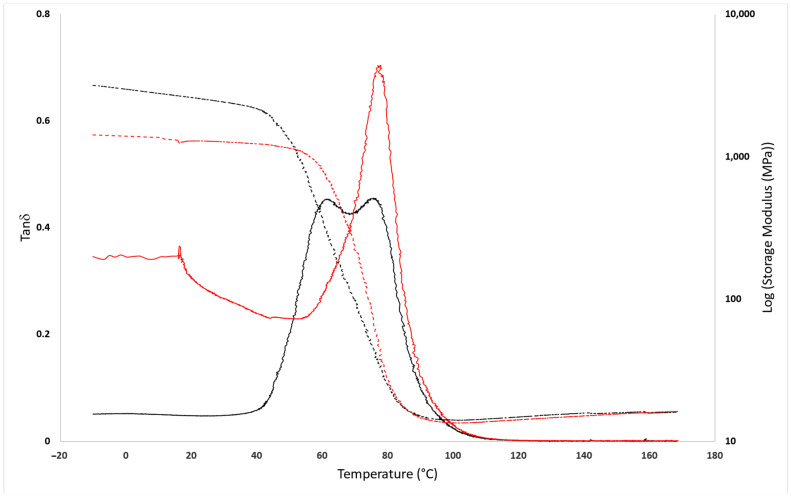
DMA analysis on the sample amine excess 5%wt FANB. Black continuous line: Tanδ of the first heating cycle; black discontinuous line: Storage Modulus of the first heating cycle; red continuous line: Tanδ of the second heating cycle; red discontinuous line: Storage Modulus of the second heating cycle.

**Figure A8:**
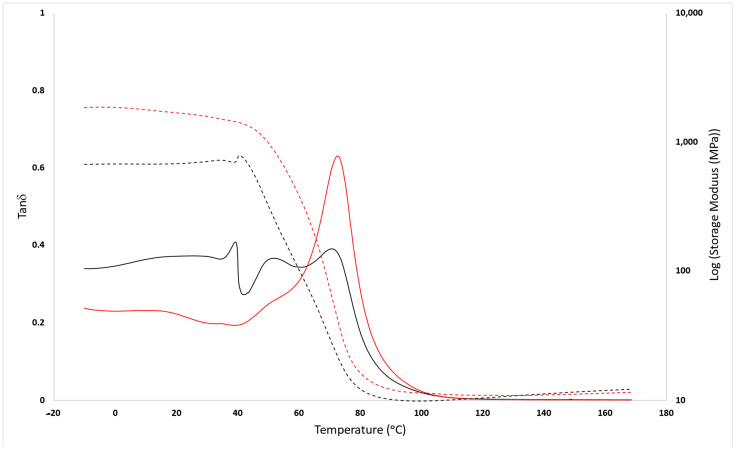
DMA analysis on the sample amine excess 15%wt FANB. Black continuous line: Tanδ of the first heating cycle; black discontinuous line: Storage Modulus of the first heating cycle; red continuous line: Tanδ of the second heating cycle; red discontinuous line: Storage Modulus of the second heating cycle.

## Data Availability

The data presented in this study are available on request from the corresponding author. The data are not publicly available due to containing information that could compromise the privacy of research participants.
